# Transcriptional profiling of the rat nucleus accumbens after modest or high alcohol exposure

**DOI:** 10.1371/journal.pone.0181084

**Published:** 2017-07-17

**Authors:** Julia Morud, Arghavan Ashouri, Erik Larsson, Mia Ericson, Bo Söderpalm

**Affiliations:** 1 Addiction Biology Unit, Department of Psychiatry and Neurochemistry, Institute of Neuroscience and Physiology, University of Gothenburg, Gothenburg, Sweden; 2 Department of Medical Biochemistry and Cell Biology, Institute of Biomedicine, University of Gothenburg, Gothenburg, Sweden; 3 Beroendekliniken, Sahlgrenska University Hospital, Gothenburg, Sweden; Radboud University Medical Centre, NETHERLANDS

## Abstract

Alcohol use disorder is a chronic relapsing brain disorder and a global health issue. Prolonged high alcohol consumption increases the risk for dependence development, a complex state that includes progressive alterations in brain function. The molecular mechanisms behind these changes remain to be fully disclosed, but several genes show altered expression in various regions of the rat brain even after modest alcohol exposure. The present study utilizes whole-transcriptome sequencing (RNA-seq) to investigate expression changes in the brain nucleus accumbens (NAc), an area of particular interest in addictive disorders, of alcohol consuming rats. The impact on gene expression after eight weeks of moderate voluntary alcohol consumption or voluntary consumption combined with forced excessive exposure was explored in two separate experiments. The results point to a lack of strong and consistent expression alterations in the NAc after alcohol exposure, suggesting that transcriptional effects of alcohol are weak or transient, or occur primarily in brain regions other than NAc.

## Introduction

Prolonged high alcohol consumption is known to increase the risk for dependence, which has been suggested to result from drug-induced molecular changes leading to gene expression alterations and cellular adaptations [1–4]. These effects are difficult to study in human subjects, and animal models, such as selected rat lines and inbred mice strains that voluntarily consume high levels of alcohol, are therefore crucial. A brain area known to be of importance for the rewarding effect of alcohol in rodents is the nucleus accumbens (NAc), the human counterpart of which repeatedly has been found altered in alcoholics [5–7].

Several studies have applied genomics in order to examine ethanol-induced transcriptional effects in NAc, as well as other brain regions, using outbred rodent alcohol models [8–11]. From these studies, it seems clear that the model applied is of importance for the outcome [12–14], as the results appear to diverge between voluntary alcohol consumption and forced intake through alcohol vapour [9, 11], as well as between the rat strain used and even between animal suppliers providing a certain strain [15, 16]. Different rat strains also display different alcohol metabolism [17]. Similarly important is the brain region studied, since certain alcohol-induced transcriptional changes only appear to occur in particular areas [8–11]

Thus, available studies have shown inconsistent results, in part due to varying ethanol exposure times and levels as well as differences in the chosen exposure model [18]. This motivates further study of transcriptional effects of alcohol consumption in the NAc. In an attempt to increase the validity of the model, we harvested tissue from animals that received excessive amounts of alcohol through gavage, in addition to voluntary alcohol consumption. The present data is another indication of the difficulties met when trying to model human alcoholism, which hinder the understanding of the disorder at the molecular and genetic levels and thereby also development of novel rational treatment alternatives.

## Material and methods

### Subjects

A total of 22 male outbred Wistar rats (Taconic, Ejby, Denmark), weighing 160-180g at the start of experiments were single housed during eight weeks, a time period previously reported to be sufficient for the detection of potential alcohol-induced alterations in gene expression [9]. Animals were housed with a regular 12h light cycle and had ad lib access to tap water and rodent chow (Lantmännen, Stockholm, Sweden). All animals were allowed one week of adaption to the animal facilities before starting experiments. Experiments were carried out in accordance with the guidelines laid down by the Swedish Animal Council and the EU directive 2010/63 regarding the care and use of animals for experimental procedures and were approved by the Ethics Committee for Animal Experiments, Gothenburg, Sweden.

### Voluntary and forced ethanol consumption

The experiments were done in two parts.

Experiment one. A total of ten animals were used, of which five animals received only water and five animals were given continuous access to 6 v/v % ethanol (EtOH) and water during an eight-week period.

Experiment two. A total of 12 animals were used of which six animals were exposed to continuous EtOH (6%) and water during a four-week period followed by three weeks EtOH gavage treatment (2.5 g/kg, 30 v/v % EtOH, twice daily) and an additional two week continuous access period. Six animals were given continuous access to 6% EtOH and water during the same period combined with three weeks of water gavage treatment.

Fluid consumption together with body weight were measured twice weekly in both experiments. At the end of the experiments animals were sacrificed and the NAc were dissected bilaterally and snap frozen. Drinking data is presented in S1 Table.

### Transcriptome sequencing and analysis

After the completion of the drinking experiments all animals were sacrificed immediately (less than 5 minutes) after removal of the EtOH bottles, in order to minimize expression alterations induced by alcohol withdrawal [19]. The NAc core and shell were dissected and RNA from 17 EtOH exposed animals (11 modest exposure and 6 high exposure) and 5 water controls were isolated with the RNeasy Lipid Tissue Mini kit (Qiagen). All RNA samples had RIN values >8 (Bioanalyzer 2100, Agilent, CA, USA). PolyA+ sequencing libraries were prepared using a non-strand specific procedure for experiment one and a strand-specific procedure for experiment two, using Illumina TruSeq reagents. Sequencing was performed using an Illumina HiSeq 2000 for experiment one, and an Illumina HiSeq 2500 for experiment two, at the Science for Life Laboratory, Stockholm. Reads had the length of 2x101 bp for the first experiment and 2x126 bp for the second experiment (additional sequencing information in S2 Table. Data was deposited in the GEO database (accession ID GSE73627). Reads were aligned to the rn6 reference genome (NCBI GenBank Assembly ID: GCA_000001895.4) using Tophat2 [20], and the number of read counts for each gene were calculated with the htseq-count tool [21] to prepare the data for differential expression analysis. Further analyses, including normalization and differential expression statistics, were performed using the DESeq2 package in R/Bioconductor [22]. Independent filtering was used for lowly expressed genes (50% threshold, corresponding to a base mean value of 43.63 in experiment one and 66.47 in experiment two). Differential expression statistics, which in DeSeq2 is based on the negative binomial distribution, was performed by comparing treated animals to controls separately within each experiment (one or two).

## Results

We determined transcriptional effects of alcohol consumption in two rat models using RNA-seq. In experiment one, animals were exposed to continuous and voluntary EtOH exposure over an eight-week long consumption period, whereas in experiment two, animals were exposed to excessive amounts of EtOH through gavage, in addition to voluntary consumption, in an attempt to increase the validity of the drinking model by increasing exposure levels. At the end of both experiments tissue from NAc was harvested and sequenced. During the drinking study in experiment one, rats voluntarily consumed in average 5.1–8.5 g/kg/day EtOH during the first four weeks, whereas the consumption in experiment two prior to gavage treatment was 2.5–4.2 g/kg/day (S1 Table). In experiment two half of the rats received additional amounts of alcohol through a gavage tube (2.5 g/kg twice daily) during three weeks.

### No genome-wide significant effect in NAc after modest or high alcohol exposure

**Fig 1:**
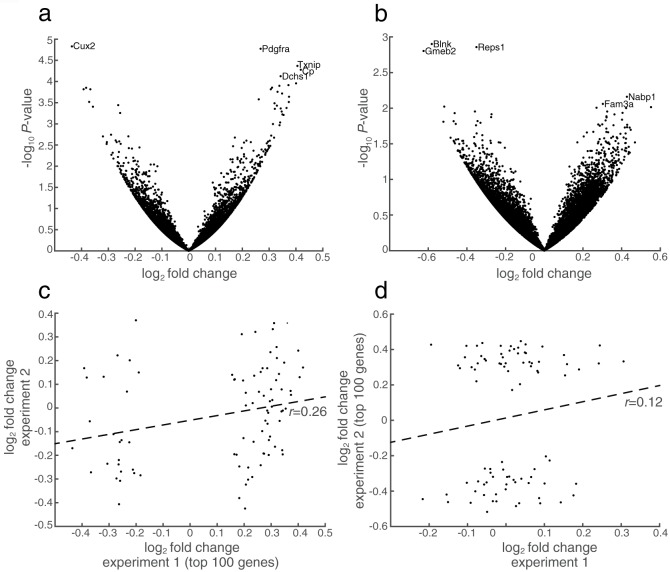
Expression changes in response to alcohol exposure. Using two different experimental setups in rats. (**a**) Volcano plot showing expression changes in experiment one; genome-wide profiling of NAc from rats exposed to continuous voluntary intake of ethanol (6%) over an eight-week period. The top 5 genes based on *P*-value are indicated with their gene name. (**b**) Volcano plot similar to panel **a**, showing expression changes in experiment two; genome-wide profiling of NAc from rats exposed to continues access of EtOH (6%) during four weeks followed by additional EtOH exposure trough gavage treatment over three weeks. (**c**) The top 100 genes were selected based on *P*-values from experiment one, and log_2_ fold changes for these genes were compared in between the two experiments. Pearson’s *r* = 0.2590, *P* = 0.0154 (**d**) Same as panel **c** but using genes selected based on *P*-values from experiment two. *r* = 0.1208, *P* = 0.2624.

The RNA-seq analysis of accumbal tissue from alcohol consuming animals revealed no genome-wide significant effects at a false discovery rate (FDR) < 0.05 in any of the experiments (Fig 1a and 1b, Table 1). In experiment one (voluntary intake), 11 genes were detected at FDR < 0.1 (S2 Table). None of these genes have previously been implicated in this context, and the results should be interpreted with care due to the weak significance.

**Table 1:** Ranking of the top ten genes with lowest *P*-value.

Experiment one				Experiment two			
Gene	RE(log_2_)	P-value	FDR	Gene	RE(log_2_)	P-value	FDR
*Cux2*	-0.436	0.00001	0.069	*Blnk*	-0.586	0.00126	0.679
*Pdgfra*	0.268	0.00002	0.069	*Reps1*	-0.351	0.00139	0.679
*Txnip*	0.405	0.00004	0.097	*Gmeb2*	-0.624	0.00157	0.679
*Cp*	0.418	0.00005	0.097	*Nabp1*	0.427	0.00691	0.679
*Dchs1*	0.343	0.00007	0.097	*Fam3a*	0.303	0.00871	0.679
*Dlx1*	0.400	0.00011	0.097	*Hsd17b8*	-0.517	0.00950	0.679
*Cd24*	0.372	0.00012	0.097	*Fancd2*	0.552	0.00967	0.679
*Etv1*	0.338	0.00013	0.097	*Cox5a*	0.270	0.00986	0.679
*Dcx*	0.311	0.00014	0.097	*Pdgfc*	0.434	0.00997	0.679
*Lgi2*	-0.383	0.00014	0.097	*Wdr92*	-0.359	0.01112	0.679

The top ten genes in each RNA-seq experiment, ranked based on *P*-values. Complete results are available in S2 Table. RE: relative expression.

### Lack of correlation in gene regulation between experiments

We next investigated whether transcriptional effects were consistent in between the two experiments, despite lack of genome-wide significant effects in the individual datasets. Interestingly, we found that expression changes for the top 100 genes from experiment one showed a weak but significant positive correlation with the same genes in experiment two (Pearson’s *r* = 0.26, *P* = 0.015; Fig 1c). This could be an indication of an overall alcohol-induced effect on these genes that is somewhat coherent in both experiments but too weak to show significance in the DeSeq2 analysis. No significant correlation was seen when comparing changes for the top 100 genes in experiment two with the corresponding genes in experiment one (*r* = 0.12, *P* = 0.26; Fig 1d).

## Discussion

Alcohol-induced transcriptomic alterations have been suggested to be involved in consolidating alcohol use disorder, but results from earlier studies have been inconsistent. There is a need for increased translatability between the human disorder and animal models of alcoholism on both a molecular and behavioural level, as this can help improve our understanding of the disease [4].

Here we employed two ethanol consumption models, both a modest voluntary exposure model and a forced model with excessive amounts of EtOH, in order to investigate the impact different exposure levels play on transcriptional changes in the NAc using RNA sequencing. Despite a fairly ambitious experimental design, including a high number of replicates and eight weeks of EtOH exposure, we did not observe any genome-wide significant effects in NAc. The experimental design thus suffers from a lack of a pure water control for experiment two, and therefore the direct comparison between the two experiments, and in the extension exposure levels, is not possible. The voluntary consumption model chosen for the current experiments often results in lower ethanol consumption levels, as could have been anticipated if using the intermittent limited access model [23]. Therefore it cannot be ruled out that the intermittent limited access model still induces chronic accumbal transcriptomic alterations. The lack of strong transcriptomic alterations in NAc is in line with other related studies in the same region [10, 24, 25], which is somewhat surprising since the NAc repeatedly is considered as one of the main regions involved in addictions, including alcoholism [26, 27]. This is indicative of region and temporal dependent molecular changes that might lead to addiction [9, 28], since numerous alterations have been found in other areas such as the prefrontal cortex and amygdala [10–12, 29].

In addition to the analysed region, it appears that the chosen drinking model has a large influence on the size of the effect. Several models have been used in earlier studies, where intermittent exposure to alcohol vapour appears to produce the most prominent alterations and to enhance ethanol self-administration the most [10, 11, 30]. This method often employs higher exposure levels that results in higher blood alcohol levels than those achieved here in experiment one (voluntary consumption), which might be required for detecting genome wide changes in gene expression. This possibility was considered in experiment two, in which forced high alcohol intake was added to voluntary intake in order to assess the effect elevated EtOH exposure plays on gene expression in NAc, however without revealing any genome-wide significances.

Notably, a weak but significant correlation was observed between fold changes observed in the two experiments, when considering the top 100 most significant genes from experiment one. This is suggestive of small but consistent transcriptional effect, and it is possible that prolonged exposure times could lead to these effects reaching genome-wide significance. In addition, several previous studies that report altered gene expression in response to EtOH have included a withdrawal period prior to measuring mRNA levels [29, 31]. This was not employed in our study, and inclusion of a withdrawal period could conceivably result in more prominent effects on gene expression in NAc. Interestingly, strong effects have been observed using similar drinking models in mice, in particular when using selected high alcohol consuming individuals [4, 32], suggestive of species differences in ethanol vulnerability.

Many important molecular changes that occur in response to alcohol consumption, such as receptor internalization and altered neural transmission [33, 34], may not be reflected in transcriptomic data and therefore may not be detected by the methods applied here. Furthermore, despite the alterations observed in the ventral striatum of alcoholics by imaging techniques [35], it is possible that transcriptomic changes are not very prominent in alcoholics either. However, the most likely cause of our negative findings may be that the animal models used fail to capture important elements of the human disease. The study thereby illustrates the difficulties met when trying to model complex human brain disorders. Unfortunately, these difficulties also hinder the mechanistic understanding of the transition from recreational drug use to sustained addiction and identification of important drug targets involved in this process.

## Supporting information

S1 Table Ethanol consumption data. EtOH and water drinking data from EtOH consuming animals for experiment one and two. Amount EtOH consumed per day and per bodyweight is included along with EtOH preference over total fluid intake.
(XLS)

S2 Table Gene list. A complete list of all annotated genes in experiment one and two, including Base Mean, log2 fold change as well as P- and FDR values. Quality control (QC) data information is included on a separate sheet.
(XLS)
